# MoS_2_ Based Photodetectors: A Review

**DOI:** 10.3390/s21082758

**Published:** 2021-04-14

**Authors:** Alberto Taffelli, Sandra Dirè, Alberto Quaranta, Lucio Pancheri

**Affiliations:** Department of Industrial Engineering, University of Trento, Via Sommarive 9, 38123 Trento, Italy; sandra.dire@unitn.it (S.D.); alberto.quaranta@unitn.it (A.Q.); lucio.pancheri@unitn.it (L.P.)

**Keywords:** MoS_2_, TMD, photodetector, heterostructure, thin film

## Abstract

Photodetectors based on transition metal dichalcogenides (TMDs) have been widely reported in the literature and molybdenum disulfide (MoS_2_) has been the most extensively explored for photodetection applications. The properties of MoS_2_, such as direct band gap transition in low dimensional structures, strong light–matter interaction and good carrier mobility, combined with the possibility of fabricating thin MoS_2_ films, have attracted interest for this material in the field of optoelectronics. In this work, MoS2-based photodetectors are reviewed in terms of their main performance metrics, namely responsivity, detectivity, response time and dark current. Although neat MoS2-based detectors already show remarkable characteristics in the visible spectral range, MoS_2_ can be advantageously coupled with other materials to further improve the detector performance Nanoparticles (NPs) and quantum dots (QDs) have been exploited in combination with MoS_2_ to boost the response of the devices in the near ultraviolet (NUV) and infrared (IR) spectral range. Moreover, heterostructures with different materials (e.g., other TMDs, Graphene) can speed up the response of the photodetectors through the creation of built-in electric fields and the faster transport of charge carriers. Finally, in order to enhance the stability of the devices, perovskites have been exploited both as passivation layers and as electron reservoirs.

## 1. Introduction

Recent improvements in optoelectronics have been partly focused on the use of two-dimensional materials to produce photodetectors. The possibility of fabricating very thin optoelectronic devices, having high performance, low production costs and mechanical flexibility has been emerging in the last decade. Graphene was the first 2D material considered for photodetection applications, thanks to its outstanding electrical properties, in particular its impressive planar mobility, reaching 200,000 cm^2^/(V s), that allows to build photodetectors with bandwidth up to 40 GHz [1,2]. However, one of its mayor limitation for its use as photodetector active layer is the absence of an energy band gap, leading to high noise contribution to the signal, arising from dark currents.

Therefore, the investigation of 2D materials with finite bandgap has increased in recent years and transition metal dichalcogenides (TMDs) have aroused more and more interest. Despite the modest mobility reported for these materials, which can reach about 200 cm^2^/(V s) [3], TMDs possess interesting electro-optical properties. A transition from indirect to direct bandgap has been observed in TMDs by reducing the dimensions from the bulk material to the monolayer limit [4]. Moreover, a strong light–matter interaction is observed for 2D-TMDs, due to the direct band gap and to the strong excitonic nature of their low dimensional structures. For TMDs, absorbance values that are one order of magnitude higher than Si and GaAs are reported [5], thus providing strong light absorption with a very thin layer of the photoactive material. These features, combined with a higher mechanical flexibility of 2D-TMDs compared to their bulk structures, allow us to fabricate very thin photodetectors also based on flexible substrates, opening the possibility to realize flexible and wearable devices. Applications for such devices can be related to medicine, biosensing, optical communications and security.

**Figure 1:**
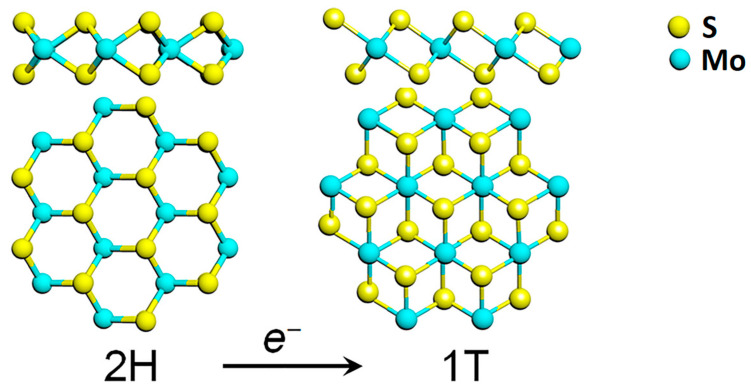
Transformation of the hexagonal 2H polymorph of MoS_2_ into its 1T phase, through electron transfer. Reprinted with permission from ref. [6]. © 2017 AIP Publishing.

Among the TMDs, molybdenum disulfide (MoS_2_) attracted much interest in the last decade, also due to the relative abundance of molybdenite in nature. MoS_2_ belongs to the family of the group VI transition metal dichalcogenides, where a layer of transition metal atoms (Mo, W) is sandwiched between two layers of chalcogen atoms (S, Se, Te) as depicted in Figure 1.

Each MoS_2_ layer is generally stacked onto each other via weak van der Waals forces in an ABA stacking sequence, building a hexagonal structure (2H-TMD), which possesses a semiconducting behaviour. Another metastable phase of MoS_2_ is known, with a tetragonal symmetry (1T-MoS_2_) and ABC stacking sequence (Figure 1). The 1T phase of MoS_2_ is not stable at room temperature [7], but it can be induced by several processes such as chemical treatment [8], plasmonic hot electron transfer [9], electron beam irradiation [6,10], through charge transfer in the TMD lattice. A subsequent annealing process is then required to restore the 2H-MoS_2_ phase [6].

**Figure 2:**
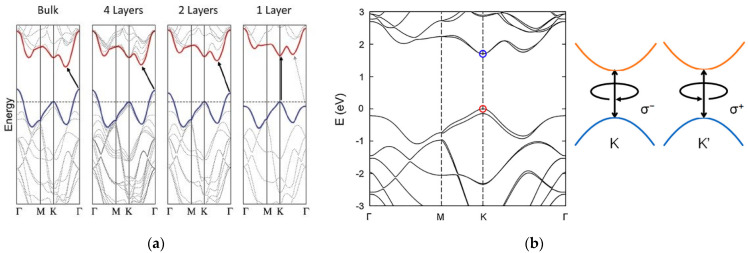
Energetic band structure of MoS_2_. (**a**) Transition from indirect to direct band gap moving from the bulk MoS_2_ to the single layer of MoS_2_. Reprinted with permission from ref. [15]. © 2010, American Chemical Society. (**b**) Degeneracy at the K point in the valence band of a single MoS_2_ layer. Reprinted with permission from ref. [6]. © 2017, AIP Publishing.

Bulk TMD electronic properties are dominated by indirect transition from the maximum of the valence band, located at the Γ point of the Brillouin zone, and the minimum of the conduction band [11,12,13]. For MoS_2_, the bulk electronic structure is characterized by an indirect energy band gap of about 1.2 eV [14]. As with other group VI TMDs, at the monolayer limit MoS_2_ modifies its energy band structure towards a direct electronic transition from the K and K’ points of the Brillouin zone, reaching an energy band gap of 1.8 eV [14,15]. This behaviour can be explained by an increase in the indirect band gap due to a considerable quantum confinement effect in the out-of-plane direction when the dimensions of the material are reduced to few layers. On the other hand, the direct transition remains unaffected, becoming the minimum energetic band-to-band transition [11,12,13] (Figure 2a). Moreover, TMDs are reported to have strong spin-orbit coupling (SOC), associated with the d-orbitals of transition metals [6,16,17]. The SOC breaks the degeneracy in the valence band, leading to two energetic maxima located at the K and K’ points, separated by an energy splitting of 160 meV for a monolayer MoS_2_ [16] (Figure 2b). This broken degeneracy opens for MoS_2_ the possibility for optoelectronic applications in the field of valleytronics [18,19].

**Figure 3:**
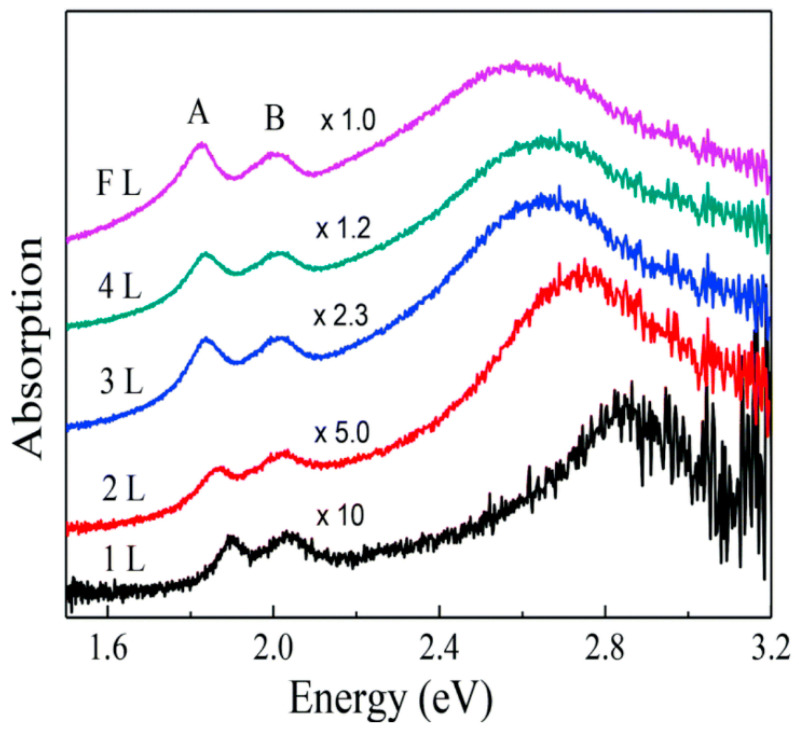
Absorption spectrum of MoS_2_ varying the number of layers from 1 layer (1L) to few layers (FL), where A and B represent the excitonic peaks of MoS_2_. Reprinted with permission from ref. [20]. 2014, Creative Commons Attribution 3.0 Unported Licence.

When MoS_2_ is irradiated with photon energies larger than its bandgap, photons are absorbed and electrons are promoted to the conduction band, leaving holes in the valence band. The optical absorption of visible light by MoS_2_ in the monolayer limit is dominated by the direct transition from the K and K’ points of the valence band. However, light absorption experiments show peculiar resonant features in 2D structures of MoS_2_ (Figure 3), that can be associated with its strong excitonic nature [4,15]. The experimentally observed absorption peaks at specific energies (EA = 1.88 eV, EB = 2.03 eV in the monolayer limit [20]) represent the excitonic energies of MoS_2_. The relative positions of the A and B peaks are related both to an increase in the SOC and to a reduction in the bandgap, approaching the bulk structure of MoS_2_ [20].

For these reasons, in 2D-MoS_2_, the bandgap measured with optical techniques turns out to be lower than the one measured with electronic techniques [6]. The strong light–matter interaction that characterizes MoS_2_ is reflected in a high absorption coefficient that can reach about 10^6^/cm [21], which is at least one order of magnitude higher than standard semiconductors like Si and GaAs. Moreover, a single MoS_2_ layer is reported to absorb up to 10% of the sunlight [5]. This superior light absorption makes MoS_2_ suitable to build photodetectors based on very thin layers of material, still having high light conversion efficiency.

Therefore, the production of thin MoS_2_ films is a central step of the device fabrication and several approaches have been investigated so far. Thanks to the weak van der Waals interaction between MoS_2_ layers, it is possible to obtain mono- or few-layered structures by a simple mechanical exfoliation of bulk MoS_2_ [22]. This approach has been extensively used in many studies [23,24,25,26,27,28,29,30,31,32,33,34,35,36] for its intrinsic simplicity, but suffers from some critical issues. In fact, MoS_2_ mechanical exfoliation generally leads to very small flakes (lateral size < 10 um) [37] and it is a low yield process. Therefore, it is unsuitable for industrially scalable-applications [38]. Other synthesis methods have been investigated to overcome these critical aspects. Chemical vapour deposition (CVD) is a powerful bottom-up approach. This method is the most compatible with the existing semiconductor technology. In CVD, large area films with high uniformity can be grown directly on the substrate, through the chemical reactions involved in the process. Despite the high controllability of the process and the high uniformity that can be achieved, CVD has the limit of being a costly process and requires high temperatures (700–1000 °C), making the process less affordable and not suitable for deposition on flexible substrates. Moreover, in most of the CVD processes used to produce MoS_2_, one of the precursors involved is H_2_S, which is toxic. As an alternative to standard CVD, plasma-enhanced CVD (PECVD) has also been exploited in order to reduce the temperature needed for the reaction (150–300 °C), thus allowing film deposition also on plastic substrates [39].

Among the bottom-up approaches, wet chemical syntheses can also be exploited for the fabrication of large area MoS_2_ films on different substrates. MoS_2_ sols can be prepared both at ambient pressure or under hydrothermal [40] or solvothermal [41] synthesis conditions. The sols can be used for coating different substrates by spin-coating or dip-coating. Generally, an annealing process (at 500–800 °C) is required in order to improve the crystallinity of the sample and an additional sulphurisation step is needed. Recently, Nardi et al. [42] have obtained MoS_2_ thin layers on Pt, SiO_2_ and flexible polyimide substrates by the sol–gel approach, using an aqueous sol prepared at ambient pressure; the coatings were annealed at low temperature (350–400 °C) without any additional sulphurisation process.

The advantages of the solution methods are represented by the versatility of the deposition technique, the low costs of production and the process scalability. The drawbacks with respect to CVD are instead the lower uniformity of the film and its minimum thickness, which is generally limited to some tens of nanometres.

The fabrication methods cited above generally lead to n-type behaviour of MoS_2_. The n-type character of pristine MoS_2_ is commonly associated to the electron donor nature of the sulphur atoms [43]. In order to exploit the full potential of MoS_2_ and to build p–n junctions, doping is required to tune the energetic levels at the interface of MoS_2_ with other materials. Doping through standard ion implantation is not suitable for 2D materials, and other methods have been investigated in the literature. The most common methods for doping MoS_2_ rely on substitutional doping and surface doping, beside the electrostatic gating. Substitutional doping consists of the substitution of a sulphur atom with an impurity within the MoS_2_ lattice. Niobium (Nb) substitutional p-doping has been reported in the literature by both chemical vapour transport (CVT) [43] and CVD [44]. Moreover, laser assisted substitutional phosphorous (P) p-doping was reported in [45] and manganese (Mn) substitutional p-doping has been investigated in [46], via a vapour phase deposition technique. Finally, p-type substitutions with fluorine (F) and oxygen (O) were obtained via plasma assisted doping by [47]. On the other hand, surface doping exploits the difference between the electron surface potential of MoS_2_ and the redox potential of the chemically adsorbed species. Nicotinamide adenine dinucleotide (NADH) has been reported to cause an n-doping to MoS_2_, while tetrafluoro-tetracyanoquinodimethane (F4-TCNQ) and 7,7,8,8-tetracyanoquinodimethane (TCNQ) have been exploited for p-doping [48]. Other molecules exploited as p-dopands for TMDs are O_2_, H_2_O and NO_2_, while potassium (K), benzyl viologen (BV) and bis(trifluoromethane) sulfonamide (TFSI) have been used for n-doping [6].

## 2. MoS_2_ Photodetectors

The strong light absorption of MoS_2_, combined with its good mobility and the possibility to fabricate very thin layers, led in the last decade to a great interest in this material for photodetection applications. In all photodetectors based on semiconductors, photons with energy larger than the material bandgap are absorbed and generate electron–hole pairs that can move under the action of an electric field. Devices may rely on different physical mechanisms for what concerns the charge drift and collection, giving rise to different photodetector categories. Most light detectors can be grouped into three classes: photoconductors, phototransistor and photodiodes. This review summarizes the different photodetector structures based on MoS_2_ presented so far.

**Figure 4:**
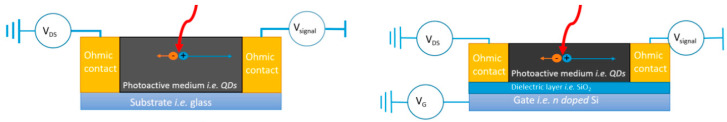
Photoconductor scheme (**left**) and phototransistor scheme (**right**). Adapted with permission from ref. [49]. Copyright 2020, Creative Commons Attribution 4.0 Unported Licence.

In photoconductors, the radiation creates electron–hole (e–h) pairs, which are then separated by an external applied bias voltage (Figure 4). The charges drift towards the electrodes where they are collected, producing a photocurrent. The mechanism beneath the signal detection is the photoconduction, namely incident photons cause an increase in the charge density and thus in the conductivity of the material. Moreover, a mechanism called photoconductive gain can be exploited in photoconductors to enhance the signal level. The gain is defined as G = τ/t, where *τ* is the lifetime of one of the charge carriers (e.g., holes) and *t* is the transit time of the opposite carriers (e.g., electrons). A gain arises when one of the charge carriers recirculate many times before it recombines with his opposite counterpart. Generally, energy states within the bandgap of the semiconductor, often induced by defects, are able to trap one of the two carriers, prolonging their lifetime and leading to multiple recirculation of the opposite carriers. The lifetime of the carriers strongly depends on the presence of trapping center within the material and can vary by several orders of magnitude, from few nanoseconds [23] to milliseconds [35]. In practice, trapping can be achieved by controlling the defects present in the material or by introducing sensitizing centres such as QDs or nanoparticles. Photoconductive gain affects the signal intensity but also its temporal response, which is governed by the carriers’ lifetime. Generally, devices relying on the photoconductive gain reach very high values of responsivity, but present slower response and consequently a lower bandwidth compared to G=1 photoconductors.

On the other hand, phototransistors are able to maximize the detector performance by reducing the noise rather than enhancing the signal intensity. In addition to the electrical contacts found in the photoconductors, here called “source” and “drain”, a third terminal (“gate”) electrically isolated from the semiconductor through a thin dielectric layer is present. Gate bias is generally exploited to deplete the semiconductor channel from carriers, in order to suppress dark current signals in the detector and thus maximize its signal-to-noise ratio (SNR). Moreover, the gate also modulates the mobility of the carriers, leading to high ON/OFF ratio values and higher values of the responsivity. Photoconductors and phototransistors unavoidably require an external power supply to sustain a voltage difference between the electrodes, which may become significant in large area detectors.

**Figure 5:**
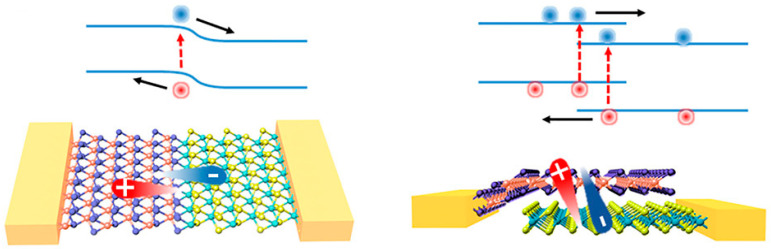
In-plane p–n junction (**left**) and out-of-plane p–n junction (**right**) of 2D materials, and their respective electronic band alignment. Reprinted with permission from ref. [6]. © 2017, AIP Publishing.

Photodiodes rely on the photovoltaic (PV) effect to collect charges. A built-in electric field is created at the junction between p- and n-sides of the semiconductor or by a Schottky barrier between a semiconductor and its metal contact. The built-in electric field can reach very high values in proximity of the junction, and thus the photogenerated carriers are driven to opposite contacts through an intrinsic voltage potential rather than an external power supply. Photodiodes can be composed of p–n junctions of the same material (homojunctions), of different materials (heterojunctions) or of metal–semiconductor junctions (Schottky diodes). Moreover, energy band alignment at the heterojunction can be exploited to suppress the drift of charges between the two sides of the junction, thus reducing the dark signals. Photodiodes can be arranged in a horizontal fashion, where two materials are put side by side (in-plane junctions), or vertically stacked, where they are put one on top of each other (out-of-plane junctions) (Figure 5).

In the next section we will discuss a selection of photodetectors based on MoS_2_ reported in the literature, chosen from among the most meaningful ones.

### 2.1. Neat MoS_2_ Photodetectors

Neat MoS2-based photoconductors are the simplest photodetectors that will be discussed in this review. They are generally composed of an insulating substrate material (e.g., SiO_2_, Al_2_O_3_, Si_3_N_4_) over which MoS_2_ is deposited. The metal contacts can be created directly on the substrate or can be deposited on the MoS_2_ surface by physical vapour deposition methods. The metal contacts can be designed properly to maximize the response and the speed of the device, as in the case of interdigitated finger contacts.

**Figure 6:**
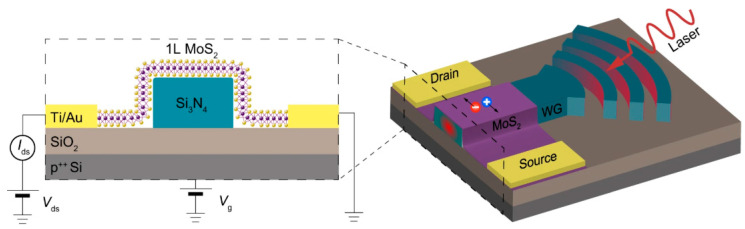
Scheme of a phototransistor and its implementation in a nanophotonic device. Reprinted with permission from ref. [27]. © 2019, Springer Nature.

Gonzalez Marin et al. [27] fabricated a phototransistor, based on a mechanically exfoliated MoS_2_ monolayer deposited on a Si/SiO_2_ substrate, which reached a large photoresponsivity (R = 10^3^ A/W) in the visible range. However, such a high response was obtained via a large photogain mechanism, arising from a very long carrier lifetime, which resulted in a response time of 13 s. Their device was also implemented into a nanophotonic circuit to test its applicability in nanophotonics (Figure 6). In fact, a single MoS_2_ layer was successfully applied over a waveguide to detect the waveguide losses.

**Figure 7:**
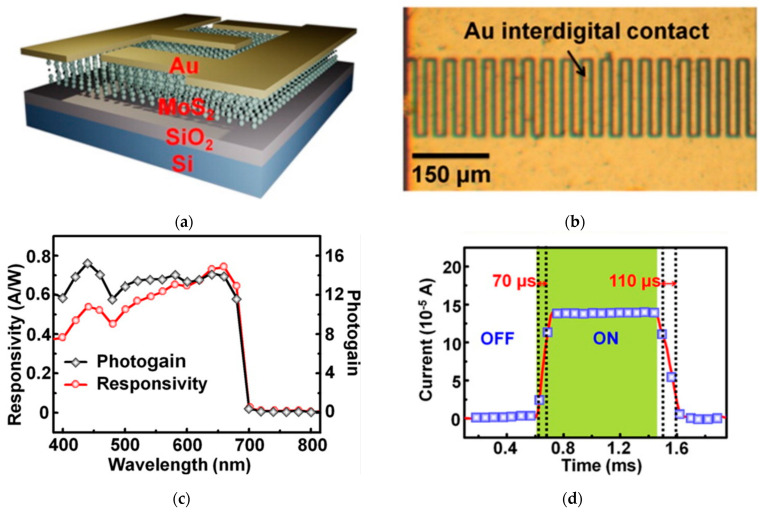
(**a**) Scheme of a photoconductor based on MoS_2_; (**b**) optical microscope top view of the device, where Au interdigitated contacts are clearly visible; (**c**) responsivity and photogain for the device over the NUV-IR spectral range; (**d**) photoswitching time response of the detector. Reprinted with permission from ref. [50]. © 2013, American Chemical Society.

A significantly faster device was produced by Tsai et al. [50], based on a few-layered MoS_2_ structure obtained through a wet-synthesis approach, and deposited on a Si/SiO_2_ substrate. The Au electrodes were fabricated by photolithography in an interdigitated fashion (Figure 7) to produce a metal–semiconductor–metal (MSM) Schottky photodiode. The detector showed very fast response to visible light (t_rise = 70 μs, t_fall = 110 μs) and a responsivity of R = 0.57 A/W. The reason for this performance is the good compromise between modest photogain and smart geometry of the device which speeds up the carrier collection. In fact, Au electrodes produced via photolitograpy in an interdigitated fashion with 8μm finger spacing (Figure 7b), play an important role in diminishing the time needed to collect the carriers.

Yore et al. [51] built an array of photodetectors based on MoS_2_ monolayers through a CVD deposition process, and achieved a response extending towards the ultraviolet spectral region (λ∼400 nm). The devices exhibited a photoresponsivity of about 1 mA/W, with a fast response, t ∼ 0.5 ms. Moreover, the detectors exhibited an extremely low dark current, I_d_ ≤ 10 fA, attributable both to a bipolar Schottky barrier between the MoS_2_ and the metal contacts, and to a negligible doping of the sample from charge impurities introduced by the substrate through an efficient deposition process.

Lopez-Sanchez et al. [24] developed one of the first phototransistors based on MoS_2_, obtained through a mechanically exfoliated single layer deposited on a Si/SiO2 substrate. The device showed a responsivity of 880 A/W in the visible range, attributable also to a strong photogain mechanism, which also affected the photoswitching time of the detector, which was about 9 s. They reported a very low value of the dark current, I_d_ = 2 pA, although it was achieved with a strong negative bias of the gate, V_g_ = −70 V.

A similar device was fabricated by A.R. Klots et al. [29] with faster response, in the order of 1 ms. The light response, although it was lower, reached 50 A/W, due to a photogain contribution of 10^3^ times.

**Figure 8:**
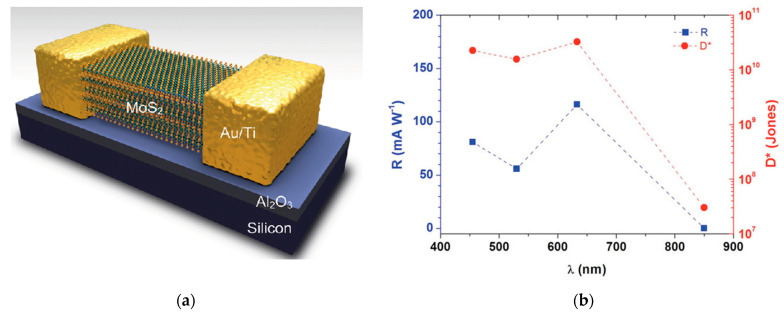
(**a**) Scheme of a phototransistor based on MoS_2_. (**b**) Responsivity and detectivity of the phototransistor over the visible-NIR spectral range. Reprinted with permission from ref. [23]. © 2012, John Wiley and Sons.

Another low-noise device is described in [23], where a multilayer MoS_2_ structure obtained through mechanical exfoliation was deposited on Al_2_O_3_ (Figure 8). The detector exhibited a responsivity of 0.12 A/W in the visible region, which dropped to very low values for near infrared radiation, attributable to the weak absorption in the IR spectral region. The detector responsivity was also affected by a short carrier lifetime τ, which was determined to be 1.27 ns, limiting the photogain mechanism. Finally, the measured dark currents were as low as 10^−11^ A. The good response, combined with the very low noise contribution of the system, led to a good detectivity of the device that reached 10^11^ Jones in the visible region. Moreover, low dark currents were achieved with a low bias of the gate terminal (Vg = −3 V).

**Figure 9:**
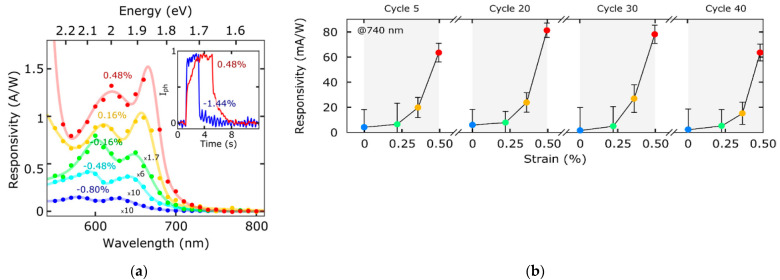
(**a**) Responsivity values over the spectral range for a ZnS-MoS2-based device when it is subject to various strains. In the inset the temporal response for different strain applications is shown. (**b**) Responsivity values against the applied strain increasing the bending cycle number. Reprinted with permission from ref. [26]. © 2019, Elsevier.

Gant et al. [26] demonstrated a strain tunable photodetector based on a single MoS_2_ layer structure, obtained by mechanical exfoliation. Their device displayed two to three orders of magnitude of variation in the responsivity when subjected to a strain in the visible range (Figure 9a). The applied strain also caused a variation of the time response from 80 ms to 1.5 s. The variation of both magnitude and time response can be attributed to the creation of trap states in the material during strain. Moreover, the measurements showed a good reproducibility when the device was tested under multiple bending cycles, demonstrating the stability of the material under strain (Figure 9b).

**Figure 10:**
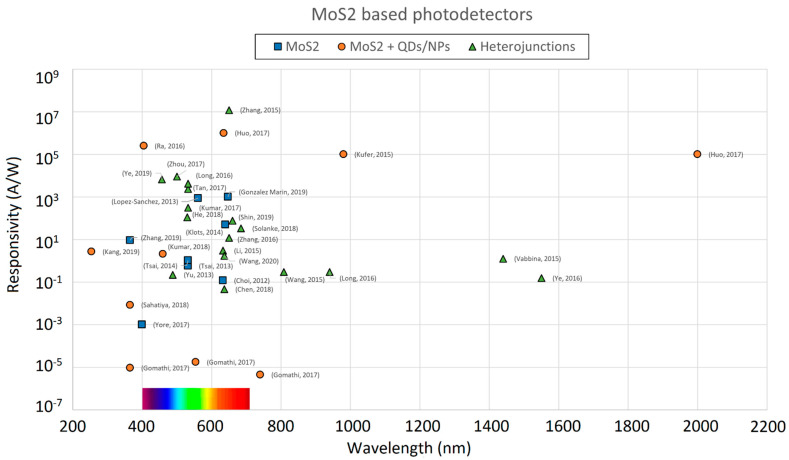
Responsivity values plotted against the wavelength for various MoS2-based photodetectors.

Neat MoS_2_ photoconductors can reach large values of responsivity when a sufficient voltage bias is applied, attributable to a large photogain contribution and can achieve relatively fast response times if the detector geometry is designed properly (e.g., with interdigitated electrodes). Most of the neat MoS_2_ photodetectors are characterized in the visible range (400–700 nm) and only very few devices explore the response to the near ultraviolet (NUV) or near infrared (NIR) spectrum (Figure 10). The IR spectrum generally leads to limited response of the material, since the absorption edge of MoS_2_ lays in the red (≈660 nm) which act as a cutoff for the longer wavelength radiation. On the contrary, the UV response is expected to increase when decreasing the wavelength, as suggested by absorption experiments.

### 2.2. MoS_2_ + QDs Based Photodetectors

To enhance the photodetector response both to NUV and near to mid infrared (NIR-MIR) light spectrum, the MoS_2_ layer is combined with sensitized centres, namely quantum dots (QDs) or nanoparticles (NPs). Devices belonging to this category generally show high responsivity values also in regions outside the visible spectrum (Figure 10), but are also characterized by slower response, caused by the trap states introduced by the sensitizing centres, which act as impurities.

**Figure 11:**
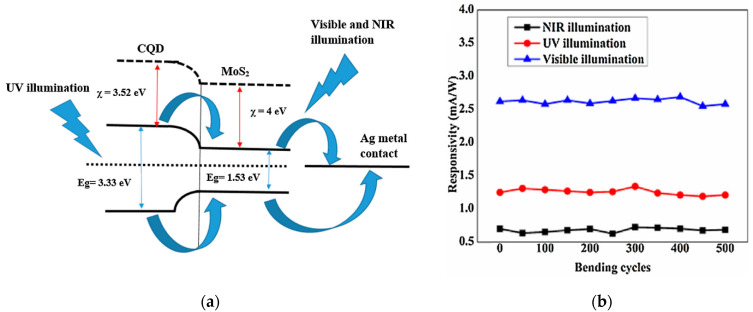
(**a**) Electronic band structure alignment for a photodetector based on MoS2 and CQDs. (**b**) Responsivity values of the device over the bending cycle numbers, demonstrating the stability of the signal over multiple bending cycles. Reprinted with permission from ref. [52]. © 2018, Elsevier.

A large area carbon QD-MoS2-based photoconductor was reported by [52] following a hydrothermal method to grow MoS_2_ on a paper substrate. The device is sensitive in the NUV with a response of 8.4 mA/W to a 365 nm light and an external quantum efficiency (EQE) of 3% for the same wavelength. Carbon QDs possess a higher band-gap compared to MoS_2_, which is suitable for UV detection (Figure 11a). A response time of 0.57 s was reported for this structure, making the device not applicable for high frequency operations. Due to the intrinsic flexibility of the paper substrate, the device was also tested under multiple bending cycles, revealing no appreciable variation of the light response (Figure 11b), thus demonstrating the possibility to build flexible and wearable electronics. As for other photoconductors, the dark current approached high values (I_d_ = 10^−6^ A), limiting the sensitivity of the device.

Similarly, a ZnS-MoS_2_ structure, deposited on paper, was fabricated by [53] and tested under UV, visible and NIR light (365 nm, 554 nm, 740 nm, respectively). The responsivity of the device in that spectral region remained orders of magnitude lower (10^−6^–10^−5^ A/W) than the previously reported values, with response times in the order of 10 s. Although the performances were low, the multiple bending test also gave a successful result in this case.

Much better performance has been obtained with phototransistors sensitized with nanoparticles. These devices require an additional power supply of tens of volts and are based on toxic compounds such as CdSe ([34]), PbS ([32]) and HgTe ([35]), but the dark current can be reduced by several orders of magnitude compared to sensitized photoconductors. Noise suppression makes these detectors highly sensitive, with detectivity values reaching 10^13^–10^15^ Jones.

**Figure 12:**
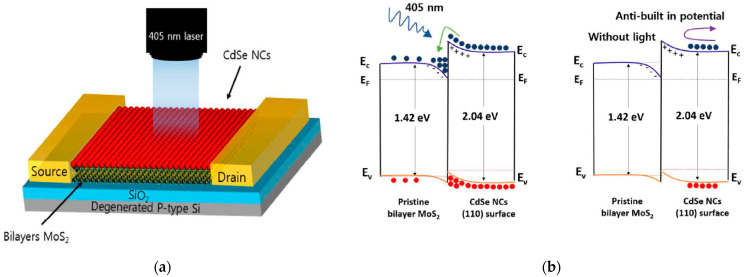
(**a**) Structure of a phototransistor sensitized with CdSe nanoparticles. (**b**) Band alignment between MoS_2_ and CdSe nanoparticles and their carrier representation when light in ON or OFF. Reprinted with permission from ref. [34]. © 2009, Royal Society of Chemistry.

A CdSe sensitized MoS_2_ phototransistor is reported in [34], based on an exfoliated MoS_2_ bilayer over a Si/SiO_2_ substrate (Figure 12). The device showed a responsivity of 2.5 × 10^5^ A/W at 405 nm of incident light, which is the highest light response of a MoS2-based photodetector in the NUV spectral range to the best of our knowledge. Although the responsivity reached very high values, the time response remained as small as 60 ms. The reasons for such performance have to be attributed to the n–n heterojunction formed by the MoS_2_ bilayer and the CdSe nanocrystals as depicted in Figure 12. In fact, when the UV radiation hits the interface region, the electrons formed in the CdSe nanocrystals are driven to the MoS_2_ side, while the holes remained confined in the CdSe nanoparticles. When the light was switched off, the electrons accumulated in the valley easily recombined and the n–n barrier prevented the transfer of carriers between the two regions.

**Figure 13:**
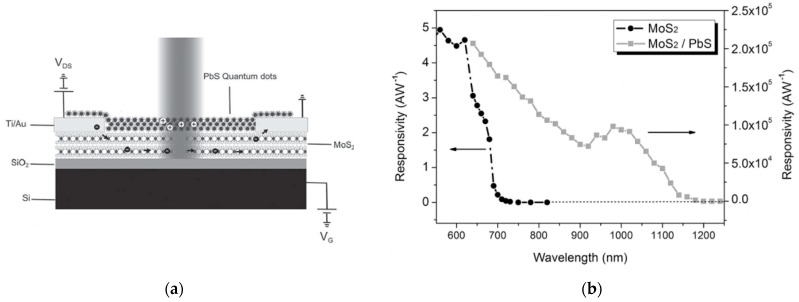
(**a**) Representation of a phototransistor based on MoS_2_ and PbS quantum dots; (**b**) Responsivity of the MoS_2_/Pbs based device compared to the values for the neat MoS2-based device over the visible-NIR spectral range. Reprinted with permission from ref. [32]. © 2014, John Wiley and Sons.

A phototransistor sensitive to the NIR light was reported in [32], composed by a few-layered MoS_2_ structure obtained through a micromechanical exfoliation, sensitized with a colloidal solution of PbS QDs (Figure 13a). This system, although its speed is limited to a subsecond time response, exhibited a response of 10^5^ A/W to a 980 nm light, and a detectivity of 7 × 10^14^ Jones. Additionally, in this case the junction (p-PbS and n-MoS_2_) plays an important role in determining the response of the device to the incident light. Moreover, the electrons are efficiently transferred from PbS QDs to MoS_2_, while the holes remain trapped in the PbS side, leading to large photogain and a slow recombination of the carriers.

The main limitation of these devices lies in the high gate bias voltage required to achieve such a high detectivity (Vg = −80 V [34], Vg = −100 V [32]), and the intrinsic toxicity of cadmium and lead. The strong gate modulation required can be attributed to the formation of the density of the states within the MoS_2_ bandgap.

**Figure 14:**
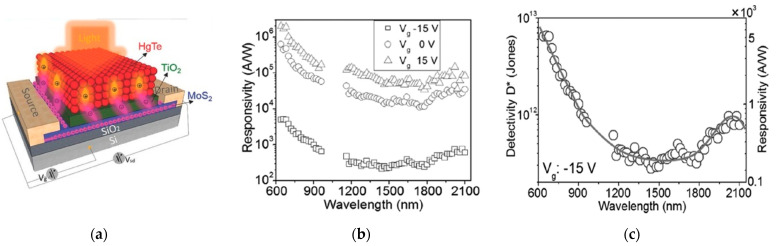
(**a**) Photodetector based on MoS_2_ + HgTe; (**b**,**c**): responsivity and detectivity values over the vis-IR spectral range, respectively, for the same phototransistor. Reprinted with permission from ref. [35]. © 2017, John Wiley and Sons.

Some efforts have been done to reduce the required gate bias, as in the MoS_2_ phototransistor sensitized with Hg-Te nanoparticles reported by [35]. The detector showed an extremely high responsivity (10^5^–10^6^ A/W) over a very broad light spectrum, ranging from visible to mid infrared (2000 nm), with a fast response time in the order of milliseconds. Such high responsivity was achieved through a high photogain mechanism, originated from a large hole lifetime (τ = 4 ms) induced by the presence of QDs, which was six orders of magnitude larger than the transit time of the electrons (t = 9 ns). The detectivity measurements showed values of about 10^12–^10^13^ Jones over the same spectral range (Figure 14), also due to the low measured value of the dark current (≈10 pA). In this device, a toxic element (Hg) is still present, but the detector was operated at a gate bias of −15 V in depletion regime, which is much lower than in previous devices. The gain in gate modulation can be attributed to the presence of a TiO_2_ buffer layer that reduces the interaction between MoS_2_ and HgTe.

**Figure 15:**
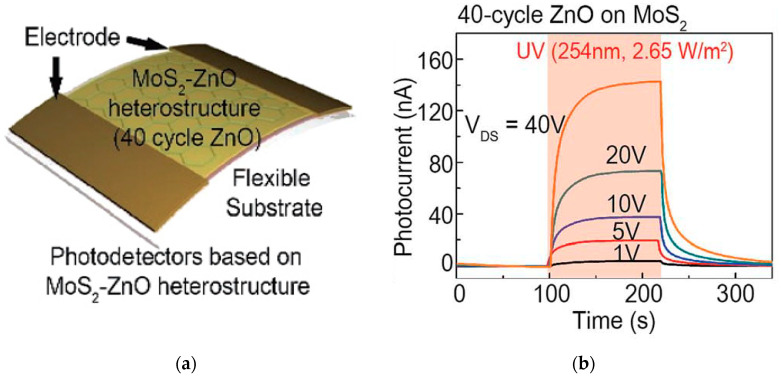
(**a**) Scheme of a phototransistor based on a MoS_2_-ZnO structure and developed on a flexible PET substrate; (**b**) response of the phototransistor to an incident UV (254 nm) light over time. Reprinted with permission from ref. [54]. Copyright 2019, Creative Commons Attribution 3.0 Unported Licence.

A more recent work [54] has explored the deep UV spectral region reporting a phototransistor based on a thin MoS_2_ film deposited on a flexible PET substrate, sensitized with ZnO nanoparticles (Figure 15a). The device showed a remarkable responsivity of 2.7 A/W to a 254 nm UV light source. Moreover, the performance was stable under multiple bending cycles. However, the response was slow, reaching tens of seconds, and the bias voltage required to obtain such a high response was set to 40 V (Figure 15b). These results make clear that more efforts have to be undertaken to produce more efficient photodetectors, especially in the deep UV light region, to broaden the possible applications of MoS_2_ at low photon wavelengths.

### 2.3. MoS_2_ + Graphene Based Photodetectors

The possibility to fabricate 2D structures of graphene and the astonishing mobility that it can reach (about 200,000 cm^2^/(V s) [55]) are some of the primary reasons for the interest in this material. However, its semimetallic behaviour, leading to a zero band gap energy, is a limiting factor in optoelectronic applications [56]. In fact, due to the absence of a forbidden band gap, large currents can be injected into the material. This leads to large dark current values, which limit the sensitivity of a photodetector. Thus, for NUV-vis-NIR application, graphene is often used as contact material [6], together with other finite band gap semiconductors. In this way it is possible to exploit the 2D versatility and high mobility of graphene by combining them with other semiconductor properties.

**Figure 16:**
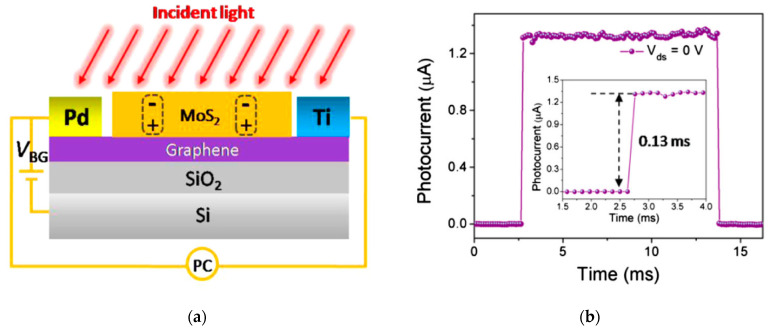
(**a**) Scheme of a phototransistor based on a graphene-MoS_2_ heterostructure. (**b**) Time-resolved response of the phototransistor (Vds = 0 V). Reprinted with permission from ref. [31]. © 2015, Elsevier.

An example of a phototransistor based on MoS_2_ with graphene is reported by [31]. The device is a lateral photodetector, where few layers of MoS_2_ were mechanically exfoliated on a sheet of graphene. Two different metal contacts (Pd and Ti) were used to collect the charges (Figure 16).

The detector exhibited a notable responsivity (R = 3 A/W) with a fast response for a lateral phototransistor (t < 0.13 ms). These results were obtained with zero voltage bias application, due to the asymmetric built-in electric field created at the graphene–electrode interface. In addition, the role of the graphene sheet is also to increase the velocity of the carrier transport to the metal contact, due to its very high mobility. Moreover, the ON/OFF ratio reached a value of 10^3^, while the dark current remained lower than 1 nA.

### 2.4. MoS_2_ + TMDs Based Photodetectors

Several studies have reported photodetectors based on heterostructures composed by different TMDs. In these devices, the different electronic band structure forms a built-in electric field at the interface between the materials that speeds up the photogenerated carrier separation. Moreover, the spectral range response of these photodetectors is widened since it involves different semiconductor materials.

**Figure 17:**
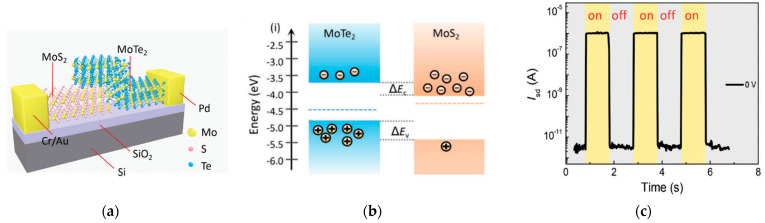
(**a**) Scheme of a photodetector based on an heterojunction between MoS_2_ and MoTe_2_. (**b**) Type II band alignment between MoTe_2_ and MoS_2_; (**c**) Photoswitching behaviour of the photodetector at V = 0 V. Reprinted with permission from ref. [36]. © 2018, John Wiley and Sons.

Chen et al. [36] proposed a photodiode based on a vertical van der Waals heterojunction between MoS_2_ and MoTe_2_, obtained through mechanical exfoliation (Figure 17a). With respect to MoS_2_, MoTe_2_ presents a smaller bandgap, which varies from 0.9 to 1.1 eV moving from bulk structure to monolayer. The structure exploits a type II band alignment, which favours the injection of electrons into MoS_2_ and the injection of holes into MoTe_2_ (Figure 17b).

The device dispalyed a responsivity of 0.046 A/W to a 637 nm light, with no voltage bias applied. Moreover, the authors measured a response time of about 60 μs, due to the fast separation of carriers that occurs at the junction. In fact, the space charge region near the junction is characterized by a very strong built-in electric field, which becomes stronger as the length of the junction is scaled down, as it happens for vertical herojunctions. The strong interlayer built-in potential provided also a barrier for the dark current, which was measured to be about 3 pA.

**Figure 18:**
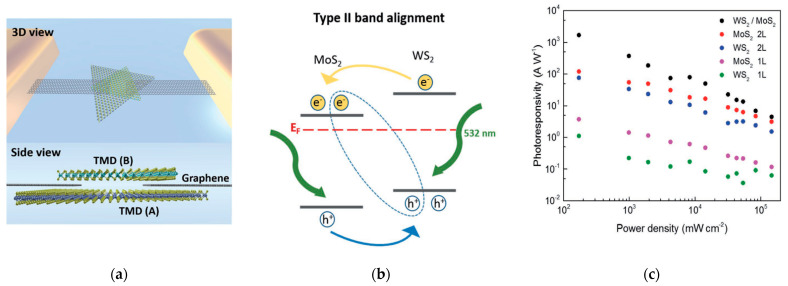
(**a**) Representation of a MoS_2_Graphene–WS_2_ heterostructure; (**b**) Band alignment at the heterojunction between MoS_2_ and WS_2_; (**c**) photoresponsivity values for different light power input of the WS_2_-Gr-MoS_2_ heterostructure (black dots), compared to the responsivity of the single TMDs. Reprinted with permission from ref. [57]. © 2017, John Wiley and Sons.

Another vertical heterostructure was reported by Tan et al. [57], who exploited a type II alignment between two monolayers of MoS_2_ and WS_2_, both obtained by CVD, with two graphene contacts (Figure 18). The detector exhibited remarkable maximum photoresponsivity (R = 2340 A/W) to 532 nm light. In particular, the photoresponsivity of the heterojunction-based device is at least one order of magnitude higher than the responsivity obtained with the single TMDs (Figure 18c). However, the decay of the response when the light was switched off lasted more than a few seconds. This performance is attributable to an efficient charge transfer at the junction and to trap states for the photogenerated carriers that lead to a photogain of about 3 × 10^4^. In fact, the measured dark current was 10^−6^ A, several orders of magnitude larger than the previously reported detector [36], due to a much less resistive channel in this case.

**Figure 19:**
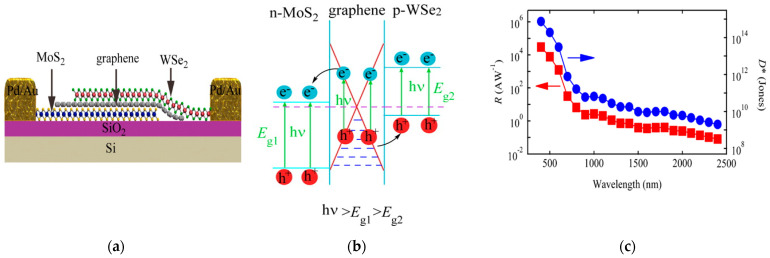
(**a)** Scheme of a phototransistor based on the vertical heterostructure between MoS_2_, graphene and MoSe_2_. (**b**) Band alignment at the heterojunction between TMDs and graphene; (**c**) responsivity and detectivity values plotted against the incident wavelength for the phototransistor over the vis-IR spectrum. Reprinted with permission from ref. [33]. © 2016, American Chemical Society.

Long et al. [33] reported on a phototransistor based on stacked MoS_2_–Graphene–WSe_2_ layers, obtained by mechanical exfoliation (Figure 19a). This device was tested over a broad light spectrum (400–2400 nm). The detector exhibited a responsivity up to 10^4^ A/W in the visible region, which decayed to 300 mA/W approaching the IR region (940 nm), with no gate bias applied (Figure 19c). The calculated specific detectivity was 10^15^ Jones in the visible region, while decayed to 10^11^ Jones in the IR region. Finally, the detector showed a time response to visible light of 54 μs, which indicates a fast response of the system, typical in a detector based on a vertical heterojunction.

**Figure 20:**
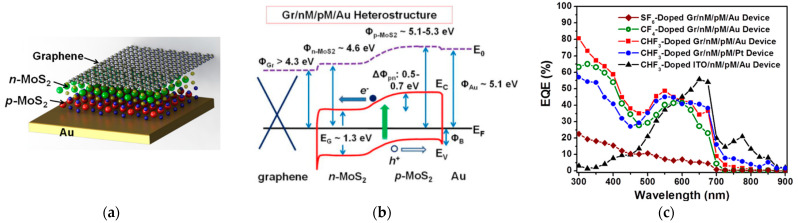
(**a**): Scheme of a photodiode based on a p–n junction of MoS_2_; (**b**) energetic band alignment between Graphene/n-MoS2/p-MoS2/Au composing the device; (**c**): external quantum efficiency (EQE) with respect to the incident wavelength for the device depicted on the left (red curve), compared with the EQE obtained with different plasma doping and different combinations of the electrode materials. Reprinted with permission from ref. [30]. © 2014, AIP Publishing.

Wi et al. [30] presented a vertical photodiode optimized for UV radiations. The structure is composed of a homojunction between a n-MoS_2_ layer and a p-MoS_2_ layer obtained sarting from exfoliation (Figure 20). p-doped MoS_2_ was obtained by CHF_3_ plasma treatment, while n-type MoS_2_ was naturally achieved through the production process. Graphene and gold were then used as contacts. The authors obtained EQE of 80.7% in the UV range (300 nm) and of 51.4 % in the visible range (532 nm).

**Figure 21:**
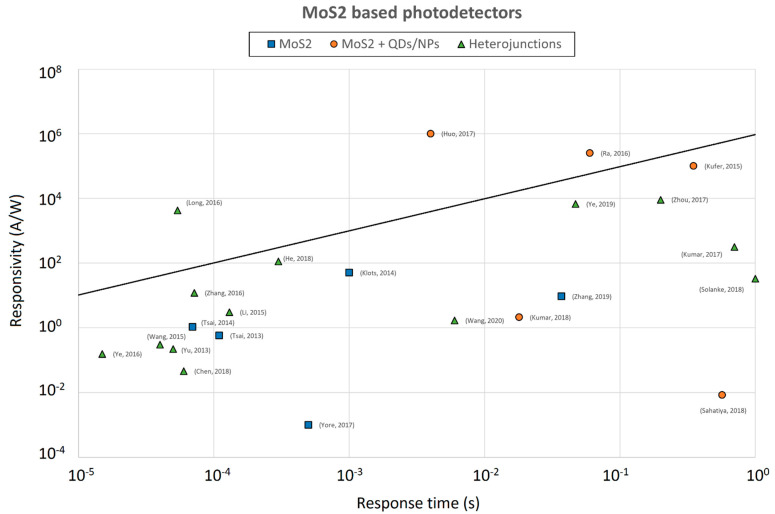
Responsivity values plotted against the response time for various MoS2-based photodetectors. The solid line represents the points for which R/τ_r_ = 1 A/ (W μs).

Both the responsivity and the response time have been plotted in Figure 21 for several MoS_2_-based photodetectors, in order to compare their performance. Since, through the photoconductive gain, the responsivity is directly proportional to the lifetime τ_r_ [1,58], a reference line representing the points for which R/τ_r_ = 1 A/(W us) has also been included in the graph. As can be observed, only three devices are represented by a point above this line.

In [33] the separation of the carriers is performed by the built in electric field at the vertical junction between the TMDs, while the transport is performed laterally by graphene, resulting in the best combination for fast carrier transport. Otherwise, devices where TMDs are responsible both for the separation of carriers and for the carrier transport may suffer from a slower response. On the other hand, MoS2-based photodetectors sensitized with QDs or NPs described in [34,35], despite their slower response compared to heterojunction beased structures, reached high performance due to an efficient carrier transfer and a remarkable photogain.

### 2.5. MoS_2_ + Perovskites Based Photodetectors

MoS_2_-based photodetectors often suffer from remarkable noise power density (NPD) and slow responses due to the defects induced on the surface of the MoS_2_ during the synthesis or from ambient gas absorption (e.g., H_2_O, O_2_) [24,59,60]. Passivation is one of the procedures exploited to reduce gas absorption at the MoS_2_ surface and the introduction of halogens have been reported to be effective on metal calchogenides [61].

**Figure 22:**
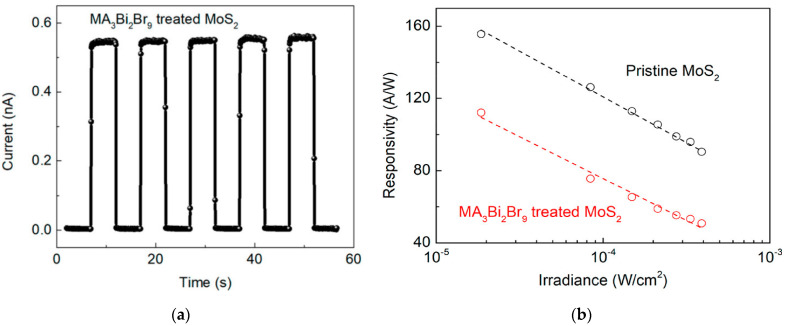
(**a**) Current measurements over time for a photoconductor based on MoS_2_ and passivated with MA_3_Bi_2_Br_9_; (**b**) Responsivity measures against irradiance for pristine MoS_2_ and MoS_2_ passivated with MA_3_Bi_2_Br_9_. Reprinted with permission from ref. [25]. © 2018, American Chemical Society.

He et al. [25] adopted a layer of methylamine (MA) halide (MA_3_Bi_2_Br_9_) to passivate a MoS_2_-based photoconductor. Their detector exhibited a high photoresponsivity (R = 112 A/W) and a very fast response (t = 0.3 ms). Moreover, the response of the device achieved a stability over the time, compared to other passivation techniques, as depicted in Figure 22.

**Figure 23:**
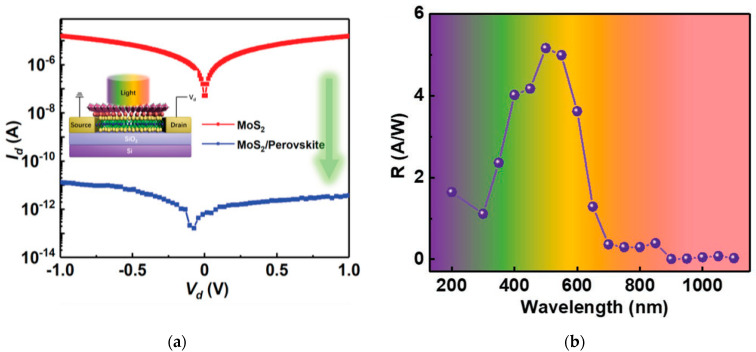
(**a**) Dark current comparison between pristine MoS_2_ and MoS_2_ passivated with (PEA)_2_PbI_4_; (**b**) Responsivity of the device with respect to the incident wavelength for a UV-vis-NIR spectrum. Reprinted with permission from ref. [28]. © 2020, John Wiley and Sons.

Recently, Wang et al. [28] have introduced a 2D halide perovskite, (C_6_H_5_C_2_H_4_NH_3_)_2_PbI_4_ ((PEA)_2_PbI_4_), over a multilayer MoS_2_ structure. They achieved a dark current suppression of six orders of magnitude with respect to the neat MoS_2_ device, reaching 10^−11^ A (Figure 23a). The reason for this behaviour is found in the electron diffusion from MoS_2_ to the perovskite: when they are put in contact, it decreases the charge carrier density. The detectivity of the device was improved as well and estimated to be about 10^13^ Jones. Moreover, the perovskite layer acts as a defect passivator, which resulted in a faster response of the device, that decreased by more than 100-fold with respect to the neat MoS_2_ case, and showed a response speed of few milliseconds. In this work, the responsivity of the detector was calculated over a broad spectral range that also included the UV spectrum (200–1100 nm) and reached maximum values in the visible range (Figure 23b).

Furthermore the device was found to operate with a zero applied external bias, due to a built-in electric field at the interface between the p-type region of the (PEA)_2_PbI_4_ and the n-type region of MoS_2_, and thus to perform as a photodiode. The response speed measured with zero voltage bias was less than one order of magnitude larger than in the case of the positive bias voltage applied, and reached few tens of milliseconds.

Many other MoS2-based devices have been reported in the literature; the performance of a few other devices ([62,63,64,65,66,67,68,69,70,71,72,73,74,75]), together with the photodetectors we have described so far, are reported in the graphs of Figure 10 and Figure 21.

## 3. Conclusions

Many photodetectors based on MoS_2_ have been developed in the last decade, and the most representative have been analyzed in this review. The main characteristics of the devices discussed so far are summarized in Table 1.

Photoconductors based on MoS_2_ are the simplest structure of photodetectors and they are able to reach very high responsivity values. On the contrary they generally suffer from a slow response, due to absence of a built-in electric field, and from high dark currents that limit the sensitivity of the device. Phototransistors based on MoS_2_ represent a more mature architecture for detecting light, due to the possibility to suppress dark current signals with an appropriate voltage bias applied at the gate terminal. Although so far very few works report the response of the MoS_2_ device to the UV and IR spectral regions, they clearly show that there is room for improvement if this material is coupled with other TMDs, graphene or quantum dots. These properties, combined with the thin structures of MoS_2_ that can be fabricated, allow us to build wide-spectrum bendable detectors.

In this direction, devices based on MoS_2_ sensitized with NPs or QDs are able to boost the response of the detectors both in the NIR-MIR and in the NUV spectrum, and are often characterized by very high responsivity values. However, the defects induced on MoS_2_ in consequence of the introduction of sensitizing centres, cause large dark current and slow response.

Heterostructures between MoS_2_ ond other materials (e.g., TMDs and graphene) are able to extend the response to a wider spectral range as well, and have been demonstrated to be suitable for the fabrication of fast photodiodes, exploiting the built-in electric field that forms at the heterointerface. Moreover, these devices are able to work also at zero-bias mode, without requiring an external power supply. The main drawback for photodiodes is instead represented by the lower responsivity that they can reach, due to the absence of the photogain mechanism. In heterostructures based on MoS_2_, the second material can act as a passivation layer, preventing the adsorption of oxygen and water molecules on the MoS_2_ surface. In this way halide perovskites have been demonstrated to be a good solution, leading to a stable and faster response of the devices.

## Figures and Tables

**Table 1:** MoS2-based photodetector performance. FL = few layers, ML = multilayer, TF = thin film, t_r_ = rise time, t_d_ = decay time.

	**Photoactive Material**	**Technology**	**MoS2 Layers**	**Wavelength (nm)**	**Responsivity (A/W)**	**Response Time (s)**	**Detectivity (Jones)**	**Dark Current (A)**	**Ref.**
**MoS_2_**	MoS_2_	phototransistor	1L	647	1000	13			[27]
	MoS_2_	MSM photodiode	FL	532	0.57	t_r_ 7 × 10^−5^ t_d_ 11 × 10^−5^	10^10^		[50]
	MoS_2_	MSM photodiode	1L	≈400	10^−3^	0.5 × 10^−3^		<10^−14^	[51]
	MoS_2_	phototransistor	1L	561	880	9		2 × 10^−12^	[24]
	MoS_2_	phototransistor	1L	640	50	10^−3^			[29]
	MoS_2_	phototransistor	ML	633	0.12		10^10^–10^11^	10^−11^	[23]
	MoS_2_	photodiode	FL	365	9.3	t_r_ 3.7 × 10^−2^ t_d_ 3.9 × 10^−2^			[62]
	MoS_2_	MSM photodiode	3L	532	1.04	t_r_ 4 × 10^−5^ t_d_ 5 × 10^−5^			[68]
**MoS_2_ + QDs/NPs**	MoS_2_ + CQD	photoconductor	FL	365	8.4 × 10^−3^	0.57		≈10^−6^	[52]
	MoS_2_ + ZnS	photoconductor	3L	554	1.79 × 10^−5^	11			[53]
				365	9.50 × 10^−6^	22			[53]
				780	4.52 × 10^−6^	31			[53]
	MoS_2_ + CdSe	phototransistor	2L	405	2.50 × 10^5^	0.06	1.24 × 10^14^		[34]
	MoS_2_ + PbS	phototransistor	FL	980	10^5^	0.35	7 × 10^14^	2.6 × 10^−7^	[32]
	MoS_2_ + HgTe	phototransistor	FL	635	10^6^			10^−11^	[35]
				2000	10^5^		10^12^		[35]
	MoS_2_ + ZnO	phototransistor	TF	254	2.7	55			[54]
**MoS_2_ Heterostructures**	MoS_2_ + Gr	phototransistor	FL	632.8	3	<1.3 × 10^−4^		9 × 10^−10^	[31]
	MoS_2_ + MoTe_2_	photodiode	FL	637	4.60 × 10^−2^	6 × 10^−5^	1.06 × 10^8^	3 × 10^−12^	[36]
	MoS_2_ + WS_2_	phototransistor	1L	532	2340		4.1 × 10^11^	10^−6^	[57]
	MoS_2_ + Gr + WSe_2_	phototransistor	1L/FL	532	4250	5.4 × 10^−5^	10^15^		[33]
				940	0.3		10^11^		[33]
	MoS_2_ + MA	photoconductor	15L	530	112	3 × 10^−4^	3.8 × 10^12^	4 × 10^−9^	[25]
	MoS_2_ + (PEA)_2_PbI_4_	photoconductor	ML	637	1.68	t_r_ 6 × 10^−3^ t_r_ 4 × 10^−3^	1.06 × 10^13^	10^−11^	[28]

## Data Availability

Data available on request.
